# Using conservation science to advance corporate biodiversity accountability

**DOI:** 10.1111/cobi.13190

**Published:** 2018-10-05

**Authors:** Prue F. E. Addison, Joseph W. Bull, E. J. Milner‐Gulland

**Affiliations:** ^1^ Department of Zoology, Interdisciplinary Centre for Conservation Science University of Oxford Oxford U.K.; ^2^ Department of Food and Resource Economics Center for Macroecology, Evolution, and Climate University of Copenhagen Copenhagen Denmark; ^3^ Durrell Institute of Conservation and Ecology, School of Anthropology and Conservation University of Kent Canterbury U.K.

**Keywords:** corporate social responsibility, development, indicators, mitigation, nature, private sector, sustainability, desarrollo, indicadores, mitigación, naturaleza, responsabilidad social corporativa, sector privado, sustentabilidad, 企业的社会责任, 可持续性, 私营部门, 发展, 减缓, 自然, 指标

## Abstract

Biodiversity declines threaten the sustainability of global economies and societies. Acknowledging this, businesses are beginning to make commitments to account for and mitigate their influence on biodiversity and report this in sustainability reports. We assessed the top 100 of the 2016 Fortune 500 Global companies' (the Fortune 100) sustainability reports to gauge the current state of corporate biodiversity accountability. Almost half (49) of the Fortune 100 mentioned biodiversity in reports, and 31 made clear biodiversity commitments, of which only 5 were specific, measureable, and time bound. A variety of biodiversity‐related activities were disclosed (e.g., managing impacts, restoring biodiversity, and investing in biodiversity), but only 9 companies provided quantitative indicators to verify the magnitude of their activities (e.g., area of habitat restored). No companies reported quantitative biodiversity outcomes, making it difficult to determine whether business actions were of sufficient magnitude to address impacts and were achieving positive outcomes for nature. Conservation science can advance approaches to corporate biodiversity accountability by helping businesses make science‐based biodiversity commitments, develop meaningful indicators, and select more targeted activities to address business impacts. With the biodiversity policy super year of 2020 rapidly approaching, now is the time for conservation scientists to engage with and support businesses in playing a critical role in setting the new agenda for a sustainable future for the planet with biodiversity at its heart.

## Abstract 2

*Article impact statement*: Science‐based biodiversity commitments, meaningful indicators, and activities need development to address environmental impacts of business.

## Abstract (es)

Uso de la Ciencia de la Conservación para Potenciar la Responsabilidad Corporativa hacia la Biodiversidad

**Resumen**

Las declinaciones de la biodiversidad amenazan a la sustentabilidad de las sociedades y economías globales. Los negocios han reconocido esto y han comenzado a comprometerse a mitigar y a responsabilizarse por su influencia sobre la biodiversidad y a reportar esto en informes sobre sustentabilidad. Evaluamos los informes sobre sustentabilidad de las 100 mejores compañías del reporte Fortune 500 Global del 2016 (el Fortune 100) para estimar el estado actual de la responsabilidad corporativa hacia la biodiversidad. Casi la mitad (49) del Fortune 100 mencionó a la biodiversidad en sus informes, y 31 dejaron claro sus compromisos con la biodiversidad, de los cuales sólo cinco fueron específicos, medibles y limitados por tiempo. Se divulgó una variedad de actividades relacionadas con la biodiversidad (p. ej.: manejo de impactos, restauración de la biodiversidad e inversión en la biodiversidad). Pero sólo nueve compañías proporcionaron indicadores cuantitativos para verificar la magnitud de sus actividades (p. ej.: área del hábitat restaurado). Ninguna compañía reportó resultados cuantitativos con respecto a la biodiversidad, lo que complica la determinación de si las acciones de las empresas fueron de una magnitud suficiente para tratar los impactos y si se están logrando resultados positivos para la naturaleza. La ciencia de la conservación puede potenciar los métodos para la responsabilidad corporativa hacia la biodiversidad ayudando a las empresas a realizar compromisos con la biodiversidad basados en la ciencia, desarrollar indicadores significativos y seleccionar actividades más enfocadas para tratar los impactos de las empresas. Con la rápida aproximación del súper año para la biodiversidad, el 2020, ahora es el momento para que los científicos de la conservación apoyen y se comprometan con las empresas para tener un papel significativo en el establecimiento de una nueva agenda con la biodiversidad como núcleo para el futuro sustentable del planeta.

## 摘要

生物多样性丧失威胁着全球经济和社会的可持续性。基于此, 企业开始承诺说明并减缓它们对生物多样性的影响, 并在可持续性发展报告中对此进行报告。我们分析了2016 年财富世界 500 强中前 100 名的企业 (即财富百强) 的可持续性报告, 以评估企业生物多样性责任制度的现状。财富百强企业中近一半 (49) 的企业在报告中提及了生物多样性, 31 个企业作出了明确的生物多样性保护承诺, 但其中只有 5 个企业的承诺具体、可测量且有时限。报告中公开了各种与生物多样性有关的活动 (如影响管理、恢复生物多样性、对生物多样性投资), 但只有 9 个企业提供了量化指标来证实这些活动的规模 (如恢复栖息地的面积) 。没有一个企业报告了定量的生物多样性保护成效, 因此很难判断这些商业行为是否足以应对对生物多样性的影响, 并正在取得积极的自然保护成效。保护科学可以通过帮助企业作出科学的生物多样性承诺、制定有意义的指标、选择更有针对性的活动来应对商业对生物多样性的影响, 从而推进企业生物多样性保护责任的发展。随着生物多样性政策年 2020 年的迫近, 保护科学家是时候参与和支持企业以生物多样性为核心的地球可持续性未来新议程的制定, 并发挥其关键作用。**【翻译: 胡怡思; 审校: 聂永刚】**

## Introduction

Biodiversity underpins and sustains ecosystems globally, and declines in biodiversity threaten the resilience of nature, global economies, and societies (Venter et al. 2016; Duffy et al. 2017). International targets exist to direct governments and inspire society to take steps toward the conservation of biodiversity in the broader context of global sustainable development (e.g., the Convention on Biological Diversity [CBD] Aichi targets [CBD 2011] and the Sustainable Development Goals [SDGs] [United Nations 2016]). The public sector has mobilized and is working toward the achievement of international targets; however, efforts to conserve biodiversity are still falling short (Butchart et al. 2010; Geldmann et al. 2013).

The international goal to “mainstream biodiversity” (CBD Strategic Goal A) (CBD 2011) sets a vision for shared responsibility across public and private sectors for the conservation of nature balanced with sustainable development (Redford et al. 2015). The mainstreaming of the biodiversity agenda has been led predominantly by the public sector, where guidance, tools, standards, and regulations have been developed to mandate and encourage the private sector to manage their impacts and dependencies on biodiversity (e.g., TEEB 2010; Forest Trends 2017). Bottom‐up signals of mainstreaming biodiversity are also emerging; companies are recognizing biodiversity loss as a risk to their operations (e.g., threatening operational productivity, access to finance, regulatory compliance, or reputation) (Dempsey 2013; Addison & Bull 2018). A public signal of businesses identifying biodiversity as a material risk is when they make commitments to or account for their influence on biodiversity in sustainability reporting (Boiral 2016).

Corporate biodiversity accountability (through external disclosure of commitments, activities, and performance) is an important aspect of organizational stewardship and legitimacy, which an increasing number of businesses are undertaking (Jones & Solomon 2013). Natural resource extraction businesses (a heavily regulated sector for impact mitigation) are increasingly making biodiversity commitments (e.g., no net loss [NNL] or better), and companies from a range of other sectors (e.g., food, financial services, and technology) are beginning to make similar commitments (e.g., to protect the environment or reduce impacts on the environment) (van Liempd & Busch 2013; Rainey et al. 2015; Adler et al. 2017). Despite these seemingly positive moves, accounting studies suggest that corporate biodiversity accountability is very much in its infancy (Jones & Solomon 2013; Boiral 2016; Adler et al. 2017).

Redford et al. (2015) suggest that conservation scientists have failed to engage with the mainstreaming‐biodiversity agenda and that there is an urgent need for a “science‐driven field of biodiversity mainstreaming,” where conservation scientists critically analyze progress to help support and improve current mainstreaming activities. In parallel, science‐based processes and tools are being called for to evaluate corporate social and environmental performance (Vörösmarty et al. 2018). A key requirement for tracking progress toward biodiversity mainstreaming is an analysis of corporate biodiversity accountability, as communicated through sustainability reports. We conducted an exploratory analysis of some of the world's largest companies to provide a snapshot of current global corporate commitments and actions for biodiversity and illustrate how conservation science could help inform more robust corporate biodiversity accountability to support the science‐driven field of biodiversity mainstreaming.

## Biodiversity Commitments and Actions of the World's Top 100 Companies

To ascertain the current status of current global commitments and actions for biodiversity, we turned to some of the world's largest companies—the Global Fortune 500. Every year *Fortune* generates an annual ranking of the largest 500 corporations worldwide, as measured by total revenue, and assesses corporate profits, assets, and employee numbers (Fortune 2016). The analysis does not include assessment of sustainability reporting. The Fortune 500 allows exploration of the extent to which companies are engaging in public disclosure of environmental and social issues and assessment of the current level of corporate biodiversity accountability.

We assessed the sustainability reports of the top 100 of the 2016 Fortune 500 Global companies (hereafter Fortune 100) (Fortune 2016) to determine how biodiversity is being integrated into business decision making and externally reported. We chose the top 100 companies in the Fortune 500 because these represent a cross‐sector of industries that are exposed to different levels of biodiversity risk (as defined by F&C 2004) through, for example, access to land, capital, or markets and relations with regulators. Based on F&C (2004) categories of biodiversity risk (Supporting Information), 31 companies are high risk (e.g., energy), 32 are medium risk (e.g., finance), and 37 are low risk (e.g., health care) sectors. We investigated which companies mention or make commitments for biodiversity; what biodiversity‐related activities are disclosed; and whether information about biodiversity activities or performance is being disclosed qualitatively or quantitatively.

Online searches for the Fortune 100 sustainability reports were conducted using the Global Reporting Initiative (GRI) sustainability disclosure database (GRI 2016*b*) (search by company name) or Google (search terms “*sustainability*” and company name). The most recent reports (dated up to 2016 and searched for in September 2017) were collated. Sustainability reports can also be referred to as environmental, corporate social responsibility, sustainability, registration reports, or financial reports that contain non‐financial information. Companies made up of multiple subsidiary companies (e.g., the Exor Group) were assessed only when sustainability reporting was done for the Fortune‐listed company as a whole, not subsidiary companies. Websites were not included in our analysis when the year of biodiversity commitments or activities were not stated; only dated interactive online sustainability reports were analyzed. Reports were searched for “*biodiversity*” OR “*nature*” OR “*species*” OR “*ecosystem*” in acknowledgment of the broad definition of *biodiversity* (CBD 2017). We also searched for terms related to biodiversity (“*forest”* OR “*palm oil”* OR “*seafood”*) that are commonly used in relation to nature‐based commodities without mention of biodiversity‐related terms.

Reports were searched for concise biodiversity commitments, which were commonly associated with a dedicated chapter or subchapter in the sustainability report or were listed as a commitment in disclosure or materiality tables of reports (e.g., Walmart: “To conserve one acre of wildlife habitat for every acre of land occupied by Walmart United States through 2015”) (Supporting Information). We evaluated corporate biodiversity goals against a subset of “Specific Measurable Assignable Realistic and Time‐related” (SMART) criteria (Doran 1981) to assess whether goals were specific (element of biodiversity the goal relates to is articulated beyond simply *biodiversity*; e.g., forest, threatened species, wetlands); measurable (quantifiable reduction or improvement is stated and a defined baseline is provided; e.g., 10% of land protected compared with 2010 levels); and time bound (goal associated with a time frame over which the company aims to achieve the goal; e.g., to achieve…by 2020).

When biodiversity was mentioned in reports, we recorded whether this was in line with the GRI (currently the most common voluntary reporting framework used for biodiversity; Boiral 2016; Boiral & Heras‐Saizarbitoria 2017) or other relevant international conventions (e.g., the SDGs biodiversity related goals 14 and 15 and the CBD). Search terms used included: “*GRI*” OR “*global reporting initiative*” OR “*sustainable development goal*” OR “*SDG*” OR “*convention on bio*” OR “*convention for bio*” OR “*CBD*.”

To assess the types of biodiversity activities undertaken by companies, reports were open‐coded to develop common themes, following an inductive category development methodology (Patton 2002). Activities were grouped into common themes once searching of all reports was complete. For each activity disclosed, we assessed whether it was described qualitatively (descriptive text provided in the sustainability report only) or quantitatively (e.g., key performance indicators or metrics are in Supporting Information).

The quantitative content analysis of all reports was undertaken by P.F.E.A., and this analysis was independently repeated by J.W.B., who coded 25% of the reports. The coders discussed the coding of the reports to assess any discrepancies. Inconsistencies were reconciled prior to data analysis, to achieve a minimum intercoder agreement of 80% (similar to methods used in recent sustainability research [e.g., Boiral & Heras‐Saizarbitoria 2017]).

### Biodiversity Mentions and Commitments

**Figure 1:**
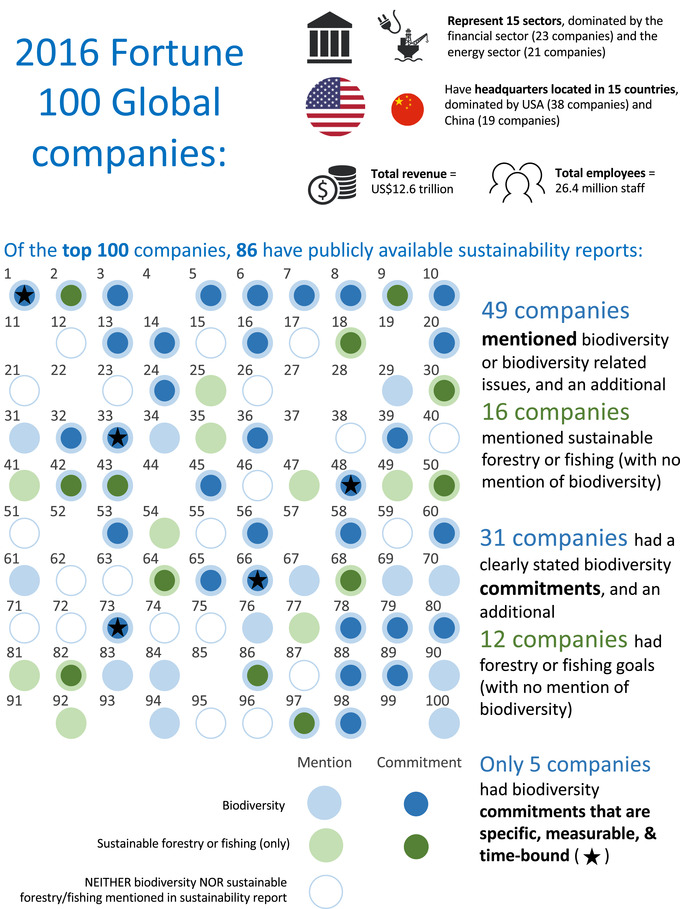
The Fortune 100 Global companies’ (with corresponding 2016 rankings) progress toward incorporating biodiversity into sustainability reporting based on mentions and commitments relating to biodiversity, sustainable forestry, or fishery in their sustainability reports (light blue, companies mentioning biodiversity; dark blue, companies with biodiversity commitments; light green, companies mentioning sustainable forestry or fishing only; dark green, companies with sustainable forestry or fishing only commitments; star, companies with specific, measurable, and time‐bound commitments). Company details are in Supporting Information and on the Fortune 500 Global website (Fortune 2016).

In 2016 the Fortune 100 represented 15 sectors and was dominated by the financial and energy sector companies (Fig. 1). Their headquarters were located in 15 countries, with over half located in the United States and China. In 2016 Fortune 100 companies employed 26.4 million people and had a total revenue of US$12.6 trillion. Sustainability reporting was undertaken by the majority of the Fortune 100 companies; 86 had publicly available sustainability reports (Fig. 1 & Supporting Information). These reports were from 2016 (74 company reports) or were the most recent reports available (2015, 7 reports; 2014, 2 reports; 2013, 2 reports; 2012, 1 report) (Supporting Information).

Almost half (49) of the Fortune 100 mentioned biodiversity or related terms, and an additional 16 companies mentioned sustainable forestry or fishing (without specifically mentioning biodiversity) (Supporting Information). Companies from higher biodiversity risk sectors did not make greater mention of biodiversity compared with lower risk sectors (companies mentioning biodiversity: high risk, 71%; medium risk, 53%; low risk, 70% [Supporting Information]). This suggests that the risk biodiversity poses to business operations is not the sole driver for inclusion of biodiversity in sustainability reports. Only 4 companies mentioned biodiversity and stated that it was not a material risk to their operations and therefore did not report on it further (BMW, HSBC Holdings, Dong Feng, and Banco Santander).

The 49 companies that mentioned biodiversity all used a typical format of sustainability disclosure, which included a predominantly qualitative narrative explaining the importance of biodiversity and what actions they take regarding biodiversity. Their treatment of biodiversity ranged from a brief single mention in the context of other environmental issues (e.g., climate change, water, and waste reduction) to a dedicated biodiversity chapter, with clear biodiversity commitments and disclosure of biodiversity‐related activities.

Twenty‐four of the 49 companies that mentioned biodiversity made links with the biodiversity‐focussed SDGs. This is far greater than the 6 companies that acknowledged the CBD. Although not intended as a reporting framework, the SDGs resonate with the private sector and are being used to frame their sustainability commitments and activities.

Only 31 of Fortune 100 companies had clearly stated commitments relating to biodiversity (Supporting Information). Commitments most commonly related to protecting biodiversity (e.g., Volkswagen: “…we promise to support the protection of species at all locations”) or to managing impacts on biodiversity (e.g., BP: “We work to avoid activities in or near protected areas and take actions to minimize and mitigate potential impacts on biodiversity”). A higher proportion of companies from high biodiversity risk sectors made biodiversity commitments compared with lower risk sectors, but unexpectedly fewer companies from medium risk sectors made biodiversity commitments compared with low risk sectors (52%, 13%, and 30% in high, medium, and low risk sectors, respectively) (Supporting Information). This pattern is attributable to so few finance companies (classed as medium risk, which include insurance, banks, and diversified financials) making biodiversity commitments (2 out of 23 companies).

Of the 23 finance sector companies, 12 were banks, and 9 of these are Equator Principles Financial Institutions (EPFIs). Eight EPFIs mentioned their adherence to the Equator Principles (which have requirements to ensure impacts on biodiversity are minimized [Equator Principles 2013]), but only 1 company had a biodiversity commitment (BNP Paribas, which commits to “combating loss of biodiversity”). Six EPFIs mentioned biodiversity, but did not translate the Equator Principles (to minimize biodiversity impacts) into a corporate commitment. One EPFI (Banco Santander) stated that biodiversity was not of material risk to them, justifying why no biodiversity information is disclosed further. The remaining 4 non‐EPFIs did not mention or make commitments for biodiversity.

Only 5 businesses (of 31) had commitments that could be classified as specific, measurable, and time bound (Walmart, Hewlett Packard, AXA, Nestlé, and Carrefour) (Fig. 1 & Supporting Information). Most of these related to commodities (e.g., Hewlett Packard: “To help protect forests, in 2016 HP set a goal to achieve zero deforestation associated with HP brand paper and paper‐based product packaging by 2020”). By contrast, the 12 of the 16 companies that made commodity commitments (but did not mention biodiversity) made specific, measurable, and time‐bound commitments (Supporting Information). The only specific, measurable, and time‐bound biodiversity commitment made was Walmart's (out of date) commitment: “To conserve one acre of wildlife habitat for every acre of land occupied by Walmart United States through 2015.” Beyond Walmart's commitment, none of the remaining Fortune 100 had adopted quantifiable biodiversity commitments (e.g., NNL or better), unlike the small but rising number of corporations outside of the Fortune 100 (Rainey et al. 2015). The lack of specific, measureable, or time‐bound features of corporate biodiversity commitments has also been observed in other recent sector‐specific and nation‐specific studies (e.g., Jones & Solomon 2013; Boiral 2016; Adler et al. 2017).

### Disclosure of Biodiversity Activities

**Figure 2:**
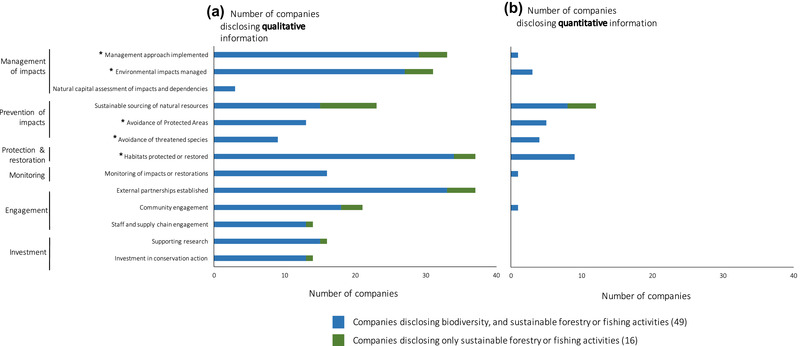
The number of companies disclosing (a) qualitative biodiversity information about activities and (b) quantitative biodiversity information about activities. Areas of disclosure include management of impacts, prevention of impacts, protection and restoration, monitoring, engagement, and investment.

The 49 companies that mentioned biodiversity and additional 16 that mentioned sustainable forestry or fishing disclosed a range of activities. Activities included managing or preventing impacts, protecting and restoring biodiversity, monitoring biodiversity, engaging and connecting people with biodiversity, and investing in biodiversity (a much greater diversity of activities than the GRI areas of biodiversity disclosure) (Fig. 2 & Supporting Information). These activities were typically described qualitatively, involving short case‐study narratives or general descriptions. Only 9 companies provided quantitative performance indicators associated with descriptions.

The lack of standardized quantitative performance indicators creates challenges for comparing performance both between companies and for individual companies through time. Although the GRI suggests performance indicators for use alongside qualitative disclosures for biodiversity, this is a voluntary framework (GRI 2016*a*), and not all businesses report this for biodiversity (26 companies reported at least 1 of the GRI areas of biodiversity disclosure).

The most commonly disclosed qualitative information concerned habitats protected or restored and partnerships formed (disclosed by 37 companies respectively) (Fig. 2). Examples of disclosed activities provided in the Supporting Information illustrate the brevity of statements made about habitats protected or restored (e.g., the reforestation of E.ON woods) and partnerships formed with NGOs and government agencies (e.g., Shell's partnerships with the International Union for Conservation of Nature [IUCN]). Other common activities included GRI biodiversity disclosure areas (GRI 2016*a*), including companies outlining the strategies or management approaches they use to manage impacts (33 companies; e.g., Société Générale follow the Equator Principles biodiversity standards) and how businesses manage their biodiversity impacts (e.g., Citigroup follow the International Finance Corporation Performance Standards by avoiding impacts on critical biodiversity habitats). Three companies discussed using natural capital assessments to help understand their impacts and dependencies on biodiversity (Walmart, Hitachi, and Nestlé) (Supporting Information). This is likely to increase in the future with the recent release of the Natural Capital Protocol, which has gained considerable traction with the private sector internationally (Natural Capital Coalition 2016).

The most commonly disclosed quantitative biodiversity information also concerned habitats protected or restored (9 companies) (Fig. 2). For example, Hitachi reported the number of ecosystem preservation activities implemented. The next most commonly cited quantitative indicator for biodiversity related to the proportion of commodities that have been sustainably sourced (e.g., Carrefour reported on the percentage increase in sales of certified seafood) (Supporting Information). Other quantitative information disclosed included the GRI areas of disclosure demonstrating the avoidance of protected areas (e.g., Glencore reported on their operations that are located in, adjacent to, or that contain protected areas) and threatened species (e.g., Enel reported on the number of IUCN Red List species affected by projects in different countries of operation), but these activities are disclosed by a very small fraction of companies, suggesting the GRI areas of biodiversity disclosure are of limited relevance to the majority of the Fortune 100. Few companies attempted to disclose quantitative information about the magnitude of their impact on biodiversity versus the magnitude of the activities they undertake, which are designed to be beneficial for biodiversity (with the exception of Glencore, which disclosed the area of impacted vs. rehabilitated land). Finally, no companies reported quantitative outcomes of their activities for biodiversity, which makes it very difficult to verify whether the implemented actions have any positive outcomes for nature.

## Informing Robust and Effective Corporate Biodiversity Accountability with Conservation Science

Our assessment of the 2016 Fortune 100 Global companies has revealed that big businesses are giving biodiversity limited treatment in sustainability reports. These empirical findings support accounting and accountability research suggesting that corporate biodiversity accountability is in its infancy (Jones & Solomon 2013; Boiral 2016; Adler et al. 2017).

This analysis has also helped identify some critical areas where conservation science could contribute to the science‐driven field of biodiversity mainstreaming (Redford et al. 2015), particularly to support more robust corporate biodiversity accountability approaches. We considered 3 critical areas where conservation science approaches, which have been successfully applied for decades to support environmental policy and management, can help businesses clarify and deepen their commitments to biodiversity and support the international biodiversity mainstreaming agenda.

### Making Science‐Based Corporate Biodiversity Commitments

Corporate biodiversity commitments are made only by a fraction of the Fortune 100, and these commitments often lack clarity (Jones & Solomon 2013; Boiral 2016) (Fig. 1). In addition, many businesses disclose information about biodiversity actions without having a clearly stated biodiversity commitment (Fig. 1). An absence of clearly defined corporate biodiversity commitments means that it is impossible to measure whether businesses are genuinely making progress in relation to managing their impacts and dependencies on biodiversity and whether they are contributing to international goals to halt the loss of biodiversity and address the underlying threats to biodiversity.

By comparison, in 2015, 80% of the world's largest 250 companies have made science‐based climate commitments and disclosed information about carbon emission reductions in their sustainability reports (KPMG 2015). The widely accepted science‐based commitments (that are specific, measurable, and time bound) used to set corporate climate commitments are a model for the general improvement of corporate biodiversity commitments. Such commitments include clearly defined aspects of climate (e.g., greenhouse gas emissions), baselines, and end dates to allow for quantitative evaluation of corporate performance. However, it is much more challenging to make science‐based biodiversity commitments. Biodiversity is a vague and complex concept, which is impossible to capture in a single or set of indicators (Purvis & Hector 2000). The CBD's definition encompasses all living things from genes to ecosystems (CBD 2017). This is where conservation science can help because many approaches have been applied successfully for decades to help set clear objectives to guide the management and measurement of biodiversity and have informed both policy and site‐level management decisions (Table 1).

**Table 1:** Examples of conservation science approaches (frameworks and modeling approaches) and their potential contribution to science‐based corporate biodiversity commitments; transparent and comparable corporate biodiversity indicators; and identification of additional avenues of corporate biodiversity action

Conservation science approach	Develop science‐based biodiversity commitments	Develop transparent and comparable biodiversity indicators	Expand and deepen corporate biodiversity action
Decision making frameworks and associated modeling techniques (e.g., structured decision making, adaptive management, and management strategy evaluation frameworks [Bunnefeld et al. 2011; Runge 2011; Addison et al. 2013; Milner‐Gulland & Shea 2017]).	Develop specific commitments relevant to business influence and impacts on biodiversity (e.g., using values‐focused thinking and conceptual models in structured decision making).	Develop indicators to evaluate corporate commitments and activities (e.g., using objectives hierarchies and conceptual models in structured decision making).	Develop actions that directly address business impacts or influence (e.g., conceptual models, consequence models, and cost‐benefit analysis in structured decision making or adaptive management). Prioritize areas for biodiversity action (e.g., systematic conservation planning). Guide evaluation and reporting on the effectiveness of biodiversity actions to contribute to corporate biodiversity commitments (e.g., using statistical models in structured decision making or adaptive management). Account for uncertainty in the effectiveness of a proposed action and help determine the magnitude of activity to be implemented (e.g., using process models within management strategy evaluation).
The mitigation hierarchy and associated principles of biodiversity management and modeling techniques (Bull et al. 2013; Arlidge et al. 2018).	Develop measurable commitments (e.g., following principles of no net loss or net positive impact). Develop meaningful spatial and temporal frames of reference for commitments (e.g., baseline or counterfactual development).	Develop indicators that can account for biodiversity gains and benefits and losses and impacts.	Guide the avoidance, minimization, restoration, and offsetting of predicted biodiversity impacts from development (i.e., applying the mitigation hierarchy). Ensure activities are new contributions to biodiversity conservation when the activity undertaken is designed to offset negative impacts (i.e., demonstrate additionality). Account for uncertainty in the effectiveness of a proposed activity and help determine the magnitude of activity to be implemented (e.g., guided by multipliers).
Protected Area Management Effectiveness Evaluation framework and associated modeling techniques (Hockings et al. 2006)	Develop specific, measurable and time‐bound commitments relevant to business influence and impacts (e.g., use conceptual models).	Develop indicators that address the full management process from inputs (resources spent), to outputs (activities undertaken), and to outcomes (changes in biodiversity).	Guide the evaluation of and reporting on the effectiveness of biodiversity activities to contribute to corporate biodiversity commitments (e.g., expert judgment, statistical models, and report cards).
SMART biodiversity commitments (Maxwell et al. 2015)	Guide the development of specific, measurable, ambitious, realistic, and time‐bound commitments.		
Essential biological variables (Pereira et al. 2013)		Identify what components of biodiversity are fundamentally important and directly under their control or influence that relate to corporate biodiversity commitments.	
Global biodiversity indicators (e.g., Butchart et al. 2010; Nicholson et al. 2012)		Develop a suite of indicators that paint a picture of pressures, biodiversity status (i.e., outcomes), and management responses to address biodiversity declines. Test the performance and sensitivity of indicators in relation to the business contexts within which they are applied.	
Composite indicator development (e.g., Burgass et al. 2017)		Develop indicators that can be aggregated from site to corporate level that account for bias and uncertainty through the aggregation process.	
International biodiversity goals, e.g., CBD Aichi targets (CBD 2011) and the Sustainable Development Goals (United Nations 2016)			Guide understanding of the types of priority biodiversity activities needed to contribute to international efforts to conserve and sustainably use biodiversity and guide more influential corporate biodiversity activity.

Decades of conservation science have reinforced the need for commitments that are specific, measurable, and time bound to guide effective conservation action (Table 1) (Brown et al. 2015; Maxwell et al. 2015). Decision‐support frameworks, such as structured decision making (Addison et al. 2013), adaptive management (Runge 2011), management strategy evaluation (Bunnefeld et al. 2011), and the mitigation hierarchy (Bull et al. 2013; Arlidge et al. 2018), can all be useful in guiding the development of science‐based corporate biodiversity commitments (Table 1). These frameworks and their associated tools can help in developing clear commitments that are specific to business influence and impacts; include quantifiable targets, accounting for both biodiversity gains and losses (e.g., NNL or better); use meaningful spatial and temporal frames of reference; and align with international strategic goals for biodiversity (Table 1) (e.g., reduce impacts, improve biodiversity status, enhance benefits to society, and support and engage in knowledge sharing [CBD 2011]).

### Developing Transparent and Comparable Corporate Biodiversity Indicators

The limited standards for corporate biodiversity disclosure mean that there are no consistent approaches to reporting biodiversity information (Fig. 2) (van Liempd & Busch 2013; Adler et al. 2017). Some businesses disclosed information about the activities they undertake to address their impacts. However, few provided details of the magnitude of these activities or quantified whether they are adequate to address the scale of the negative impacts the business is having on biodiversity (Fig. 2). In addition, few report on the outcomes of their activities for biodiversity, that is, answering the question is the biodiversity affected by the business's direct or indirect operations improving, declining, or being maintained? The general failure to report on the magnitude of negative impacts versus beneficial activities and their outcomes for biodiversity makes it enormously difficult for stakeholders and shareholders to obtain a complete and transparent view of a company's biodiversity performance and at worst could be camouflaging unsustainable business practices (Fonseca et al. 2014; Vörösmarty et al. 2018).

Conservation approaches can support the development of indicators to transparently account for biodiversity gains and losses and directly evaluate corporate commitments. Protected area management effectiveness evaluation encourages the development of indicators to address the full process of biodiversity management from inputs (resources spent) and outputs (activities undertaken) to outcomes (changes in biodiversity) (Hockings et al. 2006). Approaches used in conservation science and policy, like essential biological variables (e.g., for measures ecosystem structure or function, or species persistence [Pereira et al. 2013]), global biodiversity indicators (e.g., for measures of state, pressure, and response [Butchart et al. 2010]), and scalable composite indicators (Burgass et al. 2017) can help businesses develop indicators that support quantitative evaluation of progress toward achieving commitments. These approaches encourage careful consideration of components of biodiversity that are fundamentally important to business operations, directly under business control or influence, and development of indicators that account for both gains and losses of biodiversity. Lessons from the development of international‐level biodiversity indicators (Nicholson et al. 2012) emphasize the necessity not only to develop and implement indicators, but also to thoroughly test the performance and sensitivity of indicators in relation to the contexts within which they are applied (e.g., correct spatial and temporal resolution and sensitivity to change in response to policy and management interventions).

### Expanding and Deepening Corporate Biodiversity Action

The range of actions for biodiversity that businesses disclosed (Fig. 2) can help improve corporate social legitimacy, but may do little to genuinely address the magnitude of their environmental impacts (Jones & Solomon 2013; Boiral & Heras‐Saizarbitoria 2017). Conservation approaches can be used to target activities so that they directly address biodiversity commitments and can help businesses to predict their likely effectiveness (Table 1). Frameworks such as structured decision making, adaptive management, and management strategy evaluation and the process models used within these frameworks will help explicitly account for the uncertainties surrounding the effectiveness of activities (Milner‐Gulland & Shea 2017). The mitigation hierarchy can guide the selection of activities to mitigate impacts and create biodiversity gains (Bull et al. 2013; Arlidge et al. 2018).

Going beyond undertaking activities to account for the direct footprint of a business's impacts, a wider question is how are these activities contributing to global priorities for action to conserve biodiversity? The key international biodiversity targets (CBD Aichi Biodiversity Targets and the UN's SDGs [CBD 2011; United Nations 2016]) can, and should, be used to provide an overarching framework to guide businesses toward expanding and deepening their biodiversity activities, so that they become part of the international community, involving the public sector, civil society, and private sector that works toward a more sustainable world (Table 1).

Scientists must not underestimate the private sector's focus on risk as a reason to drive action on social and environmental issues, rather than the misconception that only companies that stand to benefit directly from the environment will take action (Addison & Bull 2018; Barbier et al. 2018). When business operations are threatened by biodiversity loss, then biodiversity becomes a material business risk. Only once this risk is quantified and realized through materiality assessment will biodiversity become more visible to the decision making departments of corporations that manage finance and risk and will be truly integrated into corporate accountability and mainstreamed through the private sector (Dempsey 2013). Our study adds to the accountability literature that shows biodiversity is yet to be consistently perceived as a material risk across the private sector (Boiral 2016; Adler et al. 2017). Advances in quantitative risk assessment are also needed to increase the visibility of biodiversity across business operations and across far more sectors to drive corporate action to halt biodiversity loss.

## Advancing Science‐Driven Biodiversity Mainstreaming

The mainstreaming biodiversity agenda is designed to engage the private sector and encourage shared responsibility for the conservation of nature balanced with sustainable development (Redford et al. 2015). Corporate biodiversity accountability—where businesses make biodiversity commitments, disclose information about biodiversity related activities, and evaluate their corporate performance in relation to their own or international biodiversity commitments—remains in its infancy (Jones & Solomon 2013; Boiral 2016; Adler et al. 2017). To genuinely contribute to the mainstreaming biodiversity agenda, businesses will need credible and robust ways to account for biodiversity throughout the supply chain that can be reported concisely at the corporate level and acted upon.

What would a more accountable business need to commit to and measure in order to demonstrate they are doing their bit for biodiversity? We believe that corporate commitments such as NNL or better for biodiversity can be applied with flexibility to target the species and ecosystems that a company impacts. Such commitments should align with existing international biodiversity policy (CBD 2011; United Nations 2016) and be couched within a global mitigation hierarchy to help shift business activities from compensatory measures (remediation, offsets) to preventative measures (avoidance, minimization of impacts [Bull et al. 2013; Arlidge et al. 2018]). Beyond objectives, quantitative measures for biodiversity outcomes are the ideal and should be specific to a company and its biodiversity risks and impacts.

What actions should a more accountable business undertake? The expertise of conservation scientists will be vital to help target corporate action where it is needed most: honing attention to operations that pose the greatest impact on biodiversity (e.g., agriculture and extractives) (Maxwell et al. 2016); directing corporate action in conservation priority areas to avoid impact to the most threatened species and ecosystems (Martin et al. 2015; Brauneder et al. 2018); and helping conserve the last wilderness areas (Watson et al. 2016).

Finally, where can conservation scientists and businesses start to tackle the complexities of business interactions with biodiversity? The approaches outlined here are all broadly applicable, but need to be tailored to ensure that biodiversity risks and impacts are captured and translated into practical advice relevant to the sector concerned. For example, some high biodiversity risk sectors, like resource extraction (oil and gas, electricity, and mining) and agriculture, have direct impacts on biodiversity and will require approaches that focus business understanding of risks and impacts at site‐level operations when developing commitments, actions, and performance measures. Other high biodiversity risk sectors, like food retailers, will require approaches that trace the biodiversity impacts of commodities through sometimes long supply chains. Finally, medium biodiversity risk sector companies, like finance and insurance firms, will require approaches that can capture indirect biodiversity impacts (e.g., through financing third parties and projects) to address biodiversity performance (e.g., through risk management).

Now is a critical time for conservation scientists to engage in order to generate a science‐driven field of biodiversity mainstreaming. Although our analysis highlights that the world's biggest businesses have a long way to go in developing and reporting on such commitments, the scene is set for rapid improvements. If these were set in place prior to the biodiversity policy super year of 2020, when the international biodiversity conservation strategy will be revisited, then businesses could truly start to play a part in the new agenda for a sustainable future for the planet, which has biodiversity at its heart.

## Supporting information

The percentage of high‐, medium‐, and low‐risk Fortune 100 Global companies with biodiversity and sustainable forestry or fishery goals by sector (Appendix S1), the 2016 Fortune 100 companies, their ranking, and their latest sustainability reports and source (Appendix S2), the 2016 Fortune 100 companies with biodiversity or biodiversity‐related commitments (Appendix S3), and examples of biodiversity‐related activities disclosed in sustainability reports by the 2016 Fortune 100 Global companies (Appendix S4) are available online. The authors are solely responsible for the content and functionality of these materials. Queries (other than absence of the material) should be directed to the corresponding author.
