# Supplementary material for: Using conservation science to advance corporate biodiversity accountability
Source: Conserv Biol. 2018 Oct 5;33(2):307–18. doi: 10.1111/cobi.13190 (PMC7379537; doi:10.1111/cobi.13190)
Supplement: Supplementary file 1 — The percentage of high‐, medium‐, and low‐risk Fortune 100 Global companies with biodiversity and sustainable forestry or fishery goals by sector (Appendix S1), the 2016 Fortune 100 companies, their ranking, and their latest sustainability reports and source (Appendix S2), the 2016 Fortune 100 companies with biodiversity or biodiversity‐related commitments (Appendix S3), and examples of biodiversity‐related activities disclosed in sustainability reports by the 2016 Fortune 100 Global companies (Appendix S4) are available online. The authors are solely responsible for the content and functionality of these materials. Queries (other than absence of the material) should be directed to the corresponding author. [file COBI-33-307-s001.docx]

# Supporting Information for *Using conservation science to advance corporate biodiversity accountability*

**Appendix S1.** The percentage of high, medium and low risk Fortune 100 Global companies with biodiversity and sustainable forestry or fishery goals by sector. The number of companies per risk category is shown in parentheses. The biodiversity risk posed to each sector, as categorized and defined by F&C (2004); see SI Table 1 for definitions.

**Appendix S2.** The 2016 Fortune 100 companies, their ranking, and their latest sustainability reports and source (websites accessed and reports downloaded during September 2017). Details of the sector that each company belongs to are provided (as defined by Fortune (2016)). The biodiversity risk posed to that sector, as categorized and defined by F&C (2004): the risks to a company that arise as a result of a situation in which biodiversity is a contributing factor (e.g., access to land, capital or markets, reputation, security of supply, relations with regulators, liabilities); high risk – most companies are likely to be exposed to biodiversity risks, and the risks are likely to be significant; medium risk - some companies are likely to be exposed to biodiversity risks; and the risks may be significant; fewer companies are likely to be exposed to biodiversity risk, and it is harder to identify how, if at all, biodiversity risks may significantly affect the companies in these sectors.

| **Fortune ranking** | **Company name** | **Sustainability Report Name and Source** | Sector | Biodiversity risk |
| --- | --- | --- | --- | --- |
| 1 | Walmart | 2016 Walmart Global Responsibility Report: http://corporate.walmart.com/2016grr | Retailing | Medium |
| 2 | State Grid | 2016 Corporate Social Responsibility Report: http://www.sgcc.com.cn/ywlm/socialresponsiility/#2016 | Energy | High |
| 3 | China National Petroleum | 2016 Corporate Social Responsibility Report: http://www.cnpc.com.cn/en/cr2016/AnnualReport_list.shtml | Energy | High |
| 4 | Sinopec Group | No sustainability report available for Sinopec Group | Energy | High |
| 5 | Royal Dutch Shell | 2016 Shell Sustainability Report: http://reports.shell.com/sustainability-report/2016/ | Energy | High |
| 6 | Exxon Mobil | 2016 Exxon Mobil Corporate Citizenship Report: http://corporate.exxonmobil.com/en/community/corporate-citizenship-report | Energy | High |
| 7 | Volkswagen | 2016 VW Sustainability Report: http://sustainabilityreport2016.volkswagenag.com/home.html | Motor Vehicles & Parts | Low |
| 8 | Toyota Motor | 2016 Sustainability Data Book: http://www.toyota-global.com/sustainability/report/sr/ | Motor Vehicles & Parts | Low |
| 9 | Apple | 2016 Environmental Responsibility Report: http://images.apple.com/environment/pdf/Apple_Environmental_Responsibility_Report_2016.pdf | Technology | Low |
| 10 | BP | 2016 Sustainability Report: http://www.bp.com/en/global/corporate/sustainability.html | Energy | High |
| 11 | Berkshire Hathaway | No sustainability report available for Berkshire Hathaway | Financials | Medium |
| 12 | McKesson | 2016 Corporate Social Responsibility Website with 2016 figures: http://mckessoncorporatecitizenship.com/our-environment/ | Wholesalers | Medium |
| 13 | Samsung Electronics | 2016 Sustainability Report: http://www.samsung.com/us/aboutsamsung/sustainability/sustainabilityreports/download/2016/2016-samsung-sustainability-report.pdf | Technology | Low |
| 14 | Glencore | 2016 Sustainability Report: http://www.glencore.com/sustainability/ | Energy | High |
| 15 | Industrial & Commercial Bank of China | 2016 Corporate Social Responsibility Report: http://www.icbc-ltd.com/ICBCLtd/SocialResponsibility/Corporate%20Social%20Responsibility/2016/ | Financials | Medium |
| 16 | Daimler | 2016 Sustainability report: https://www.daimler.com/sustainability/sustainability-report.html | Motor Vehicles & Parts | Low |
| 17 | UnitedHealth Group | 2016 Social Responsibility website (with 2016 figures): http://www.unitedhealthgroup.com/SocialResponsibility/Environment.aspx | Health Care | Low |
| 18 | CVS Health | 2015 Corporate Social Responsibility Report: http://cvshealth.com/sites/default/files/2015-csr-report.pdf | Food & Drug Stores | High |
| 19 | EXOR Group | No Sustainability Report available for EXOR group (only 2 of the 3 subsidiaries; therefore excluded from the analysis) | Financials | Medium |
| 20 | General Motors | 2016 Sustainability Report (http://www.gmsustainability.com/) | Motor Vehicles & Parts | Low |
| 21 | Ford Motor | 2016 Sustainability Report: http://database.globalreporting.org/reports/43815/ | Motor Vehicles & Parts | Low |
| 22 | China Construction Bank | Sustainability Report only available in Chinese (therefore not included in analysis): http://database.globalreporting.org/reports/50608/ | Financials | Medium |
| 23 | AT&T | 2016 Sustainability Report 2016: http://about.att.com/content/csr/home/sustainability-reporting.html | Telecommunications | Low |
| 24 | Total | 2016 Registration Document: www.total.com/sites/default/files/atoms/files/ddr2016_va_web.pdf | Energy | High |
| 25 | Hon Hai Precision Industry | 2016 Hon Hai & Foxconn Social and Environmental Responsibility Report: http://www.foxconn.com/Files/index/Foxconn_SER_en_2016.pdf | Technology | Low |
| 26 | General Electric | 2016 Integrated Summary Report & 2016 Sustainability Results Report: http://www.gesustainability.com/downloads/ | Industrials | Low |
| 27 | China State Construction Engineering | No sustainability report to download, only webpage with no date details (therefore excluded from analysis) | Engineering & Construction | High |
| 28 | AmerisourceBergen | No sustainability report to download, only webpage with no date details (therefore excluded from analysis) | Health Care | Low |
| 29 | Agricultural Bank of China | 2016 Corporate Social Responsibility Report: http://www.abchina.com/en/AboutUs/csr-report/ | Financials | Medium |
| 30 | Verizon | 2016 Corporate Responsibility Supplement: http://www.verizon.com/about/responsibility/sustainability | Telecommunications | Low |
| 31 | Chevron | 2016 Corporate Responsibility Report: https://www.chevron.com/corporate-responsibility | Energy | High |
| 32 | E.ON | 2016 Sustainability Report: http://www.eon.com/en/sustainability/environment.html | Energy | High |
| 33 | AXA | 2016 Registration Document: https://www.axa.com/en/about-us/environmental-commitments | Financials | Medium |
| 34 | Allianz | 2016 Allianz Sustainability Report: https://www.allianz.com/en/sustainability/publications/ | Financials | Medium |
| 35 | Bank of China | 2016 Corporate Social Responsibility Report: http://www.bochk.com/en/aboutus/csr/report.html | Financials | Medium |
| 36 | Honda Motor | 2016 Honda Sustainability Report: http://world.honda.com/sustainability/report/ | Motor Vehicles & Parts | Low |
| 37 | Japan Post Holdings | No sustainability report to download, only webpage with no date details (therefore excluded from analysis) | Financials | Medium |
| 38 | Costco | 2015 Costco Wholesale Sustainability Report: http://phx.corporate-ir.net/phoenix.zhtml?c=83830&p=irol-sustainability | Retailing | Medium |
| 39 | BNP Paribas | 2016 Registration Document and Financial Report: https://invest.bnpparibas.com/en/registration-documents-annual-financial-reports | Financials | Medium |
| 40 | Fannie Mae | 2014 Year in Review: http://database.globalreporting.org/reports/26033/ | Financials | Medium |
| 41 | Ping An Insurance | 2016 Corporate Social Responsibility Report: http://www.pingan.cn/en/csr/social-responsibility-report.shtml | Financials | Medium |
| 42 | Kroger | 2016 Corporate Social Responsibility Report: http://sustainability.kroger.com/pdfs/kroger-2016-csr.pdf | Food & Drug Stores | High |
| 43 | Société Générale | 2016 Corporate Social Responsibility Report: https://www.unglobalcompact.org/participation/report/cop/create-and-submit/active/238731 | Financials | Medium |
| 44 | Amazon.com | No sustainability report to download, only webpage with no date details (therefore excluded from analysis) | Technology | Low |
| 45 | China Mobile Communications | 2016 Corporate Social Responsibility Report: www.chinamobileltd.com/en/ir/reports/ar2016/sd2016.pdf | Telecommunications | Low |
| 46 | SAIC Motor | 2016 Annual Report: http://www.saicmotor.com/english/investor_relations/annual_report/index.shtml | Motor Vehicles & Parts | Low |
| 47 | Walgreens Boots Alliance | 2016 Corporate Social Responsibility Report: http://www.walgreensbootsalliance.com/corporate-social-responsibility-report/ | Food & Drug Stores | High |
| 48 | HP | 2016 Sustainability Report: http://www8.hp.com/us/en/hp-information/global-citizenship/index.html?jumpid=in_r138_us/en/corp/sustainability/main-menu-sustainability | Technology | Low |
| 49 | Assicurazioni Generali | 2016 Sustainability Report: https://www.generali.com/our-responsibilities/how-we-report | Financials | Medium |
| 50 | Cardinal Health | 2016 Environmental Sustainability Report: http://www.cardinalhealth.com/en/about-us/corporate-citizenship/environmental-sustainability.html | Health Care | Low |
| 51 | BMW | 2016 BMW Group Sustainable Value Report: https://www.bmwgroup.com/en/responsibility/sustainable-value-report.html | Motor Vehicles & Parts | Low |
| 52 | Express Scripts Holding | No sustainability report available for Express Scripts Holding | Health Care | Low |
| 53 | Nissan Motor | 2016 Sustainability Report: http://www.nissan-global.com/EN/CSR/SR/2016/ | Motor Vehicles & Parts | Low |
| 54 | China Life Insurance | 2016 Corporate Social Responsibility Report: http://www.e-chinalife.com/IRchannel/http/en/responsibility.html | Financials | Medium |
| 55 | J.P. Morgan Chase | 2016 Corporate Responsibility Report: https://www.jpmorganchase.com/corporate/Corporate-Responsibility/cr-newsletters.htm 2016 Environmental Social and Governance Report: https://www.jpmorganchase.com/corporate/About-JPMC/esg.htm | Financials | Medium |
| 56 | Gazprom | 2016 Sustainable Development Report: http://www.gazprom-neft.com/social/reports/ | Energy | High |
| 57 | China Railway Engineering | No sustainability report available for China Railway Engineering | Engineering & Construction | High |
| 58 | Petrobras | 2016 Sustainability Report: http://www.petrobras.com.br/en/society-and-environment/sustainability-report/ | Energy | High |
| 59 | Trafigura Group | 2016 Responsibility Report: https://www.trafigura.com/responsibility/2016-responsibility-report/ | Wholesalers | Medium |
| 60 | Nippon Telegraph & Telephone | 2015 NTT Group Sustainability Report: http://www.ntt.co.jp/csr_e/pdf/sustainability_report_2015e.pdf | Telecommunications | Low |
| 61 | Boeing | 2016 Environment Report: http://www.boeing.com/resources/boeingdotcom/principles/environment/pdf/2016_environment_report.pdf | Aerospace & Defense | Low |
| 62 | China Railway Construction | 2016 Annual Report: http://english.crcc.cn/art/2017/4/27/art_460_103179.html | Engineering & Construction | High |
| 63 | Microsoft | 2015 Citizenship Report: https://www.microsoft.com/about/csr/transparencyhub/citizenship-reporting/ | Technology | Low |
| 64 | Bank of America Corp. | 2016 Environmental, Social & Governance Report: https://about.bankofamerica.com/en-us/what-guides-us/environmental-sustainability.html#fbid=Tbv7w4t1C8M | Financials | Medium |
| 65 | ENI | 2016 Sustainability Report: https://www.eni.com/en_IT/sustainability/reporting.page | Energy | High |
| 66 | Nestlé | 2016 Corporate Social Responsibility Report: http://www.nestle.co.uk/csv2015/environmental-sustainability | Food, Beverages & Tobacco | High |
| 67 | Wells Fargo | 2015 Corporate Social Responsibility Report: https://www.wellsfargo.com/about/corporate-responsibility/goals-and-reporting/ | Financials | Medium |
| 68 | HSBC Holdings | 2016 Corporate Social Responsibility Report: http://www.hsbc.com/our-approach/sustainability/sustainability-quick-read | Financials | Medium |
| 69 | Home Depot | 2015 Sustainability Report: http://porchlightatl.com/The-Home-Depot-2015-Sustainability-Report/ | Retailing | Medium |
| 70 | Citigroup | 2016 Global Citizenship Report: http://www.citigroup.com/citi/news/2017/170424a.htm | Financials | Medium |
| 71 | Siemens | 2016 Sustainability Information Report: https://www.siemens.com/global/en/home/company/sustainability.html | Industrials | Low |
| 72 | Tesco | 2016 Annual Report and Financial Statements: https://www.tescoplc.com/investors/reports-results-and-presentations/reports-archive/ | Food & Drug Stores | High |
| 73 | Carrefour | 2016 Annual Financial Report: http://database.globalreporting.org/reports/51037/ | Food & Drug Stores | High |
| 74 | Phillips 66 | 2016 Sustainability Report: http://www.phillips66.com/EN/susdev/Documents/brochure/sustainability-brochure.html | Energy | High |
| 75 | Banco Santander | 2016 Sustainability Report: https://www.santander.com/csgs/Satellite/CFWCSancomQP01/en_GB/Corporate/Sustainability.html | Financials | Medium |
| 76 | Lukoil | 2014 Sustainability Report: http://www.lukoil.com/InvestorAndShareholderCenter/ReportsAndPresentations/SustainabilityReport | Energy | High |
| 77 | Crédit Agricole | 2016 Sustainability Report: http://credit-agricole.publispeak.com/corporate-social-responsibility-report-2015-2016/#/pageNumber=1 | Financials | Medium |
| 78 | Enel | 2016 Sustainability Report: http://database.globalreporting.org/reports/48325/ | Energy | High |
| 79 | Hitachi | 2016 Sustainability Report: http://www.hitachi.com/csr/download/ | Industrials | Low |
| 80 | Électricité de France | 2016 Reference Document: https://www.edf.fr/en/the-edf-group/dedicated-sections/investors-shareholders/financial-information/regulated-information/reference-documents | Energy | High |
| 81 | Dongfeng Motor Group | 2013 Social Responsibility Report: http://www.dfmc.com.cn/info/files/DFMC2013SHZRNB.pdf | Motor Vehicles & Parts | Low |
| 82 | IBM | 2016 Corporate Social Responsibility Report: https://www.ibm.com/ibm/responsibility/2016/ | Technology | Low |
| 83 | Valero Energy | 2016 Social Responsibility Report: https://www.valero.com/en-us/AboutValero/CorporateResponsibility | Energy | High |
| 84 | Hyundai Motor | 2016 Corporate Social Responsibility Report: http://csr.hyundai.com/upfile/report/sar/Sustainability_Report_en_2016.pdf | Motor Vehicles & Parts | Low |
| 85 | Anthem | No Sustainability report available, only on website with no associated dates (therefore excluded from analysis) | Health Care | Low |
| 86 | Procter & Gamble | 2016 Sustainability Report: https://us.pg.com/sustainability/at-a-glance/sustainability-reports | Household Products | Medium |
| 87 | Robert Bosch | 2016 Sustainability Report: https://www.bosch.com/sustainability/reporting-and-data/ | Motor Vehicles & Parts | Low |
| 88 | BASF | 2015 Economic, environmental and social performance: http://bericht.basf.com/2015/en/servicepages/downloads/files/BASF_Report_2015.pdf | Chemicals | Medium |
| 89 | Engie | 2016 Integrated Report: http://library.engie.com/uid_a0a18290-8825-4f9f-8785-a628c72c0ed3/data/en/pdf/full/POD_ENGIE_rapportintegre2016_EN_rev01_bd.pdf | Energy | High |
| 90 | Deutsche Telekom | 2016 Corporate Responsibility report: https://www.cr-report.telekom.com/site17/ | Telecommunications | Low |
| 91 | China Resources National | No sustainability report available for China Resources National | Retailing | Medium |
| 92 | SoftBank Group | 2016 Corporate Social Responsibility report: http://www.softbank.jp/en/corp/csr/about/reports/ | Telecommunications | Low |
| 93 | State Farm Insurance Cos. | No sustainability report to download, only webpage with no date details (therefore excluded from analysis) | Financials | Medium |
| 94 | Alphabet | 2016 Google Environmental Report: https://abc.xyz/investor/other/sustainability-and-related-information.html | Technology | Low |
| 95 | China Southern Power Grid | 2012 Corporate Social Responsibility Report (most recent English version): http://eng.csg.cn/Social_Responsibility/Social_Responsibility_Report/ | Energy | High |
| 96 | Comcast | 2016 Corporate Social Responsibility Report: http://corporate.comcast.com/images/2016-Corporate-Social-Responsibility-Report.pdf | Telecommunications | Low |
| 97 | Target | 2016 Target Corporate Social Responsibility Report: https://corporate.target.com/corporate-responsibility/ | Retailing | Medium |
| 98 | Pemex | 2013 Sustainability Report (latest available in English): http://www.pemex.com/en/responsibility/sustainable/reports/Documents/IRS_2013_eng.pdf | Energy | High |
| 99 | Pacific Construction Group | No sustainability report available for Pacific Construction Group | Engineering & Construction | High |
| 100 | Airbus Group | 2016 Registration Document: www.total.com/sites/default/files/atoms/files/ddr2016_va_web.pdf | Aerospace & Defense | Low |

**Appendix S3. The 2016 Fortune 100 companies with clearly stated biodiversity, or biodiversity related (e.g., forestry, palm oil, or seafood) commitments.**

| **2016 rank** | **Company Name** | **Commitments made relating to biodiversity (solely biodiversity or biodiversity and forestry/fisheries) or forestry/fisheries (where biodiversity is not mentioned [indicated with a *]) commitment** | **Source** |
| --- | --- | --- | --- |
| 1 | Walmart | Conserving land: “To conserve one acre of wildlife habitat for every acre of land occupied by Walmart U.S. through 2015."  *Getting to zero net deforestation: "By December 31, 2015, any palm oil used in Walmart private brand products around the world must be sustainably sourced, in accordance with the principles of the Roundtable on Sustainable Palm Oil (RSPO).” *Getting to zero net deforestation: “Source only sustainable beef that is free of Amazon deforestation by the end of 2015.” | 2016 Walmart Global Responsibility Report |
| 2 | State Grid | *Climate commitment: "Increase forest reserve volume and carbon sinks, and by 2030 China's forest reserve should be increased by 4.5 billion cubic meters than in 2005" | 2016 Corporate Social Responsibility Report |
| 3 | China National Petroleum | Conservation of Biodiversity and Natural Habitats: "We are devoted to reducing the potential influence on ecological environment and biodiversity during production and operation, and take full precautions to avoid environmental impact and work hard to restore the environment to its original state in case of any adverse impact." | 2016 Corporate Social Responsibility Report |
| 5 | Royal Dutch Shell | GOAL 14: LIFE BELOW WATER: "Shell is working with governments, non-governmental organisations and other experts to find ways to protect marine biodiversity. We aim to avoid impacts on biodiversity when developing new projects. We carry out impact assessments to minimise the extent to which local biodiversity and communities might be affected by operations. Shell is also involved in research programmes to help increase understanding of marine mammals.: GOAL 15: LIFE ON LAND: "We aim to minimise the impact our operations may have on natural environments and on people near our projects. This includes any impacts on local communities’ health, safety and access to fresh water, food or income. Our standards help reduce any impact our operations may have in areas that are rich in biodiversity or under environmental protection. We work with conservation organisations to restore natural habitats and ecosystems close to our operations. We also support rigorous sustainability standards to help ensure that our biofuels come from sustainable sources." | 2016 Shell Sustainability Report |
| 6 | Exxon Mobil | Biodiversity and ecosystem services: "Safeguarding the ability of the environment to support biodiversity and provide ecosystem services is a priority for ExxonMobil." | 2016 Exxon Mobil Corporate Citizenship Report |
| 7 | Volkswagen | Biodiversity: "Volkswagen has been committed to protecting biodiversity since 2007 and is a founder member of the Biodiversity in Good Company initiative. In our mission statement, we promise to support the protection of species at all locations" | 2016 VW Sustainability Report |
| 8 | Toyota Motor | Environmental Initiatives: "Cognizant of the importance of biodiversity and based on the Guiding Principles at Toyota, we are addressing biodiversity issues in areas such as the automobile and housing businesses, new businesses, and contributing to resolving social issues with the aim of realizing enhanced quality of the environment and prosperous societies, and achieving sustainable development." | 2016 Sustainability Data Book |
| 9 | Apple | *Forestry: "We’re not just protecting forests. We’re protecting generations of them.... That’s why we’re determined to protect and create enough responsibly managed forests around the world to cover all our packaging needs and produce fibre for future generations." | 2016 Environmental Responsibility Report |
| 10 | BP | Biodiversity and sensitive areas: "We work to avoid activities in or near protected areas and take actions to minimize and mitigate potential impacts on biodiversity" | 2016 Sustainability Report |
| 13 | Samsung Electronics | Conservation of Biodiversity: "Our Belief - Samsung Electronics recognizes the benefits and influence of the ecosystem and biodiversity. We are committed to minimizing the negative impacts on biodiversity and promoting the activities to conserve the ecosystem." | 2016 Sustainability Report |
| 14 | Glencore | Managing and protecting biodiversity: "We manage over 19,000ha of high value biodiversity offset areas. These areas protect a diverse range of threatened vegetation communities and plants, as well as providing important habitat for threatened fauna… Biodiversity and land management plans are required at all our assets. These include measures for flora and fauna conservation, weed and pest control, fire management and grazing management. Our plans allow for the continuation of grazing and other agricultural activities wherever possible." Land management: rehabilitating mining areas: "We are committed to rehabilitating and restoring the land progressively during the life of our mines, as well as when our mining activities have ceased. We aim to return mined land to either self-sustaining native ecosystems, agricultural use or other purposes agreed by our host governments in consultation with local communities." | 2016 Sustainability Report |
| 16 | Daimler | Nature conservation: "We share the responsibility for preserving the diversity of natural habitats for future generations. That is why we have been supporting the projects and initiatives of environmental organizations around the world for many years now. In this way, we contribute to making sure the earth remains a place worth living in." | 2016 Sustainability report |
| 18 | CVS Health | *Sustainable paper sourcing: "In 2015 we established a target to procure 100% of paper stock from sustainable sources by 2020" *Palm Oil: "Our goal is to ensure that by 2020, 100% of the palm oil we use in our products will come from verified, responsible sources delivered through fully traceable supply chains" | 2015 Corporate Social Responsibility Report |
| 20 | General Motors | SUPPORT BIODIVERSITY AT GM SITES: GM lands as resources and for partnerships to help address pressing global biodiversity issues. Our work reduces GM’s environmental footprint while increasing our positive handprint in the communities where we operate. Goals include achieving and improving upon Wildlife Habitat Council certification at every GM site; aligning GM’s Wildlife Habitat Council programs with regional habitat plans, country conservation goals and other relevant issues of concern; and working beyond minimum certification standards. * SOURCE SUSTAINABLE RUBBER: "GM is helping drive tire manufacturers toward net-zero deforestation and upholding human and labor rights by making an industry-first commitment to source sustainable natural rubber in our tires." | 2016 Sustainability Report (http |
| 24 | Total | Protecting biodiversity and ecosystems: "TOTAL “is committed to managing (...) its use of natural resources and its impact on biodiversity” and ecosystems in the biodiversity approach, set within the Group’s environmental framework, which incorporates the following core principles for action:  1. deploy the mitigation hierarchy “avoid – mitigate – compensate”: TOTAL applies this approach for the duration of its projects’ lifecycle to minimize the impact of its activities on biodiversity, 2. take into consideration the sensitivity of ecosystems: In the course of its business, TOTAL identifies and takes into account the diversity and sensitivity of various environments in terms of biodiversity, 3. manage biodiversity: TOTAL incorporates the biodiversity impact and risk management into its environmental management systems and refers to good practices within the industry, 4. report: TOTAL reports to its stakeholders on its biodiversity performance, and 5. improve knowledge of biodiversity: TOTAL participates in the improvement of knowledge of biodiversity and ecosystems as well as managing the stakes involved, through R&D initiatives taken with local and international partners, professional associations and the Total Foundation. | 2016 Registration Document |
| 30 | Verizon | *"Paper: "We are committed to the sustainable sourcing and use of paper, as detailed in our Paper Sourcing and Use policy." | 2016 Corporate Responsibility Supplement |
| 32 | E.ON | Environment: "As an energy company, we play a key role in climate protection. This is because the production and use of conventional energy produces large amounts of greenhouse gases. We publish our CO2 footprint and provide explicit reporting on what measures we are taking to reduce it. As part of our environmental management programmes, we also take into consideration what possible impacts our operations may have on the environment and biodiversity. Our goal is to reduce our resource and energy consumption and see to it that our plants and grids do not pose a threat to species and habitat diversity." | 2016 Sustainability Report |
| 33 | AXA | PAPER CONSUMPTION: "Forests act as a natural carbon sink, as well as a key biodiversity habitat. Certain unsustainable paper production processes threaten many natural forests. AXA signed the pledge on the “We Mean Business” (www.wemeanbusinesscoalition.org) in 2015 platform to remove commodity-driven deforestation from its supply chains by 2020. In line with this commitment, AXA implemented a policy to procure 95% of its paper from sustainable and recycled sources." | 2016 Registration Document |
| 36 | Honda Motor | Biodiversity Conservation" "We recognize, under the Honda’s Environment Statement, that biodiversity conservation initiatives are an essential part of our commitment to the preservation of the global environment. We will continue to work toward harmony between this commitment and our activities"... "We believe that minimizing the environmental impact resulting from the products we manufacture and our business activities represents the greatest contribution we can make to biodiversity conservation. " | 2016 Honda Sustainability Report |
| 39 | BNP Paribas | COMBATING LOSS OF BIODIVERSITY AND MANAGING WATER CONSUMPTION: "BNP Paribas helps to combat the loss of biodiversity in two ways:  - by taking measures to protect biodiversity. For example, in 1985, BNP Paribas Fortis bought the Virelles pond and transferred its management to three associations with a long-term lease of 99 years. Today, this 125 hectare lake is one of the largest “natural” bodies of water in Wallonia. Developed for tourism activities during the twentieth century, the concrete banks were rather unwelcoming for the reserve’s fauna and flora. A complete remodelling with natural banks enhances the “edge effect” (lagoons, islands, steep banks) and has led to a rapid increase in the diversity of species recorded. Today, the lake has a nature centre, a bird-watching centre and a hospital for birds and mammals. - by opting for responsibly sourced paper (made from pulp derived from recycled paper or from sustainably managed forests – PEFC or FSC eco-labels), BNP Paribas helps to protect forest ecosystems and their biodiversity." * Measures to combat deforestation: "BNP Paribas committed to working actively with the companies in the “Consumer Goods Forum” to help them achieve net zero deforestation in their supply chain by 2020" | 2016 Registration Document and Financial Report |
| 42 | Kroger | * Gentle on the Planet: "At Kroger, we continue to develop products that meet the needs of our customers. We recognize our customer’s interest in purchasing sustainably sourced products and the use of recyclable packaging whenever possible. As a result, Kroger’s Simple Truth bath tissue and paper towels are Forest Stewardship Council (FSC) and Rainforest Alliance Certified. In addition, the tissue is made from 100 percent recycled paper with a minimum 60 percent post-consumer recycled content. The inner corrugate tube can be recycled in U.S. curbside recycling programs and the plastic overwrap can be brought back to our stores and recycled in our in-store plastic bag recycling program."  *Wild-caught seafood: "Our new commitment now includes all wildcaught species and states that, by 2020, Kroger will source: • 100% of its wild-caught seafood from fisheries that are Marine Stewardship Council (MSC) certified, in MSC full assessment, in comprehensive Fishery Improvement Projects (FIPs), or certified by other GSSI‐recognized programs; and • Kroger will preferentially source MSC-certified wildcaught seafood and, by 2020, Kroger will source at least 90% of its volume from fisheries that are MSC certified." *Palm Oil: "We committed to purchase 100% certified sustainable palm oil (CSPO) to be used in these products by the end of 2015." | 2016 Corporate Social Responsibility Report |
| 43 | Société Générale | *ENVIRONMENTAL AND SOCIAL GENERAL GUIDELINES: " Société Générale has committed to an overall reduction in its coal-related activities (consistent with the IEA’s 2°C scenario) and, starting now, will no longer be involved in financing coal-fired power plant projects in high-income OECD countries or financing coal mine development projects. Société Générale is also reinforcing its commitment to the fight against deforestation by adopting the Soft Commodities Compact of the Banking Environment Initiative." | 2016 Corporate Social Responsibility Report |
| 45 | China Mobile Communications | Biodiversity: Why is This Material? “To intensify the construction and management of natural reserves and to increase protection of typical ecosystems, species, genes and landscape diversity” is explicitly presented in China’s 13th Five-Year Plan. In order to meet demand on communication network in various regions, we will unavoidably carry out construction and maintenance of mobile base stations in natural reserves or their adjacent areas. It is our significant responsibility to protect biodiversity in natural reserves." | 2016 Corporate Social Responsibility Report |
| 48 | HP | Our zero deforestation commitment: "Healthy, well-managed forests play a critical role in absorbing carbon dioxide and supporting biodiversity and local livelihoods. To help protect forests, in 2016 HP set a goal to achieve zero deforestation associated with HP brand paper and paper-based product packaging by 2020." (Biodiversity (GRI biodiversity disclosure G4-EN12) is explicitly linked to this Paper goal) | 2016 Sustainability Report |
| 50 | Cardinal Health | *Renewable energy: "The Cardinal Health Rayong, Thailand manufacturing operation, with a long standing commitment toward environmental sustainability, can operate a biomass renewable energy boiler depending on economic conditions. Fueled by sustainable eucalyptus wood wastes, the renewable energy generator is capable of offsetting 100% of the natural gas consumption in the manufacturing process. The biomass boiler utilizes fuel supplied by partners that are certified Forest Stewardship Council (FSC) 100%. (04)" | 2016 Environmental Sustainability Report |
| 53 | Nissan Motor | Air, Water, Soil, Biodiversity: "Humankind depends upon balanced ecosystems encompassing air, water, soil and living creatures. To maintain our irreplaceable world in a healthy state for future generations, Nissan is working to minimize its impact on ecosystems through its corporate activities and the lifecycle of its vehicles, making this approach a new part of its values as it continues to develop and champion environmentally friendly technologies." | 2016 Sustainability Report |
| 56 | Gazprom | MITIGATING NEGATIVE ENVIRONMENTAL IMPACTS AND THE EFFECTIVE USE OF RESOURCES: "The Company’s strategic goals in ensuring environmental safety and the sustainable use of natural resources are: to preserve biodiversity amidst conditions of a growing man-made burden" | 2016 Sustainable Development Report |
| 58 | Petrobras | Biodiversity: "The spatial distribution and diversity of our operations result in a frequent interface with protected and sensitive areas. The identification of such areas aims at a step to prevent and mitigate associated impacts and risks. " | 2016 Sustainability Report |
| 60 | Nippon Telegraph & Telephone (NTT) | Green Vision 2020: Conserving Biodiversity, targets for 2020: “Implementation based on our business activities”; "Monitor the impact of our business activities and continuously promote conservation initiatives"; “Implementation based on social contribution”; "Promote conservation initiatives with stakeholders" | 2015 NTT Group Sustainability Report |
| 64 | Bank of America Corp. | * Paper: "Paper is a significant contributor to our environmental footprint. We have three paper-usage goals we’ve worked to achieve by 2015: decrease our overall paper use by 20 percent; use an average of 20 percent post-consumer recycled content by weight; and use only paper sourced from certified forests" | 2016 Environmental, Social & Governance Report |
| 65 | ENI | Biodiversity and Ecosystem Services: "Eni’s commitment to Biodiversity and Ecosystem Services (BES) is an integral part of the Company’s sustainability policy published in 2011. In line with this policy Eni operates according to internationally recognized best practices in order to protect biodiversity and the ecosystem services from the first exploration stages to the end of the project cycle. Principles in line with the Convention of Biological Diversity (CBD), the Energy and Biodiversity Initiative’s guidelines and the tools developed by IPIECA and IOGP are applied. Moreover, Eni is working with UNEP-WCMC to publish an ad hoc policy on biodiversity and ecosystem services." | 2016 Sustainability Report |
| 66 | Nestlé | Preserve natural capital, including forests: "We recognise that our long-term success depends upon natural capital, particularly forests, land, soils and water. We aim to develop our business and operations to safeguard natural capital, acting as a responsible steward of natural resources, particularly in terms of water preservation, net zero deforestation by 2020, soil management and biodiversity conservation." *Deforestation: "Deforestation is a major environmental issue associated with palm oil production. Poor forest management and the loss of high conservation value (HCV) areas remain a challenge. Rising consumer demand means tropical rainforests and associated peatlands have been cleared to make way for plantations, which contribute to greenhouse gas emissions, soil erosion, water pollution and a loss of biodiversity. In 2010, Nestlé made a ‘no deforestation’ commitment, stating that all of its products, globally, will not be associated with deforestation by 2020 (we also support the Consumer Goods Forum’s ambition for zero net deforestation by 2020). This commitment was the first of its kind by a food company, and covers all the raw materials we use to make our foods and beverages, as well as our packaging. *Fish and seafood: "We understand the importance of having sustainable fisheries and healthy fish farms, as well as the immense challenges we all must overcome to source fish and seafood responsibly. Therefore, we work closely with our suppliers to identify, as far as possible, the sources of our fish and seafood ingredients. Our ambition is to confirm that the fish and seafood we source come from healthy fisheries or farms engaged in improvement projects." | 2016 Corporate Social Responsibility Report |
| 68 | HSBC Holdings | * Sustainable operations: "Our paper sourcing policy continues to ensure the paper we use for our own purposes meets the same standards we expect of our customers under our Forestry Policy." | 2016 Corporate Social Responsibility Report |
| 73 | Carrefour | The action plan to protect biodiversity: "Developing agroecology, organic products and fair trade (The Group is committed to integrating the principles of agroecology into the manufacture of products in its Carrefour Quality Lines and to distributing organic, fair trade products. Its aim is to offer quality products over the long term while protecting biodiversity)"; "Encouraging sustainable consumption of seafood (by 2020, 50% of Carrefour seafood products sold in the fresh and frozen sections (fished or farmed) must come from responsible suppliers)"; "Encouraging sourcing of deforestation-free products - (Carrefour is targeting zero deforestation by 2020)"; "Developing apiculture (Aware of the crucial role played by pollination in guaranteeing food security and diversity, Carrefour is lending its support to the development of apiculture. Since 2011, the Group has installed integrate biodiversity at its sites and support local beekeepers in all beehives on the roofs of its stores or in the surrounding area to countries of operation.)" | 2016 Annual Financial Report |
| 78 | Enel | Environmental policy: "Achieving the ideal insertion of industrial plant and buildings in the local area, while protecting biodiversity" | 2016 Sustainability Report |
| 79 | Hitachi | Preserving Ecosystems: "Hitachi believes that preserving ecosystems for diverse living organisms is vital to ensuring that biodiversity—a precious asset—will remain intact for future generations. We are reducing the burden on the ecosystem from the perspective of the entire life cycle of products and striving to produce things in a way that does not damage the ecosystem. We are also promoting Group-wide activities that contribute to preserving ecosystems." | 2016 Sustainability Report |
| 80 | Électricité de France | Environmental quality approach: "to launch a positive approach to biodiversity, not limited to understanding and reducing the impacts of our activities in the long run but having a positive effect on biodiversity" | 2016 Reference Document |
| 82 | IBM | *Sourcing of paper and paper/wood-based packaging materials: "IBM established its voluntary environmental goal for the responsible sourcing of paper and paper/wood-based packaging in 2002. It required that the paper and paper/wood-based packaging directly acquired by IBM be procured from suppliers that source from sustainably managed forests, where such sources exist....In 2016, the goal was enhanced requiring suppliers either to disclose sources for paper/wood to IBM, or provide evidence that sources have been certified to be from sustainably managed forests by an accredited third-party certification scheme." | 2016 Corporate Social Responsibility Report |
| 86 | Procter & Gamble | *"Responsible Forestry: "Our efforts are based on our commitment to ensure the responsible management of the world’s forests and our conscientious use of forest products. Although we do not own or manage commercial forests, many of our products and packages are dependent on raw materials from the forest-based supply chains. Therefore, we recognize the key role we must play through our procurement and manufacturing practices to ensure the sustainability of the world’s forest resources. Wood pulp for tissue/towel and absorbent hygiene products, wood fibers used in paper packaging, and palm oil for our laundry and beauty products are strategic commodities where our sourcing practices can have the greatest impact on ensuring responsible use of the world’s forest resources. Each of these materials has a different supply chain, and their unique complexities led us to develop individual programs as described in the following sections." *Responsible Forestry — Wood Pulp Supply Chains: "We give preference to pulp from Forest Stewardship Council® (FSC®) certified forests when it is available and meets product performance and business requirements. FSC protects water, wildlife and local people by ensuring forests are responsibly managed. FSC standards are recognized worldwide as the most-stringent forest management certification scheme and is supported by internationally recognized environmental NGOs" *Responsible Forestry — Palm Oil: "We are committed to ensuring that our sourcing of palm oil, palm kernel oil and derivatives does not contribute to deforestation and respects the rights of workers and indigenous peoples. We are members of the Roundtable on Sustainable Palm Oil (RSPO) and support their standards to drive responsible palm practices across the industry. In addition, we take steps beyond RSPO certification to confirm the palm-derived materials we purchase are not contributing to deforestation."" *Palm oil and palm oil fractions: "Establish traceability to palm oil mills by December 31, 2015 (Achieved)"; " "Suppliers to submit plans by December 31, 2015, to ensure no deforestation in their supply chain by 2020 (Achieved)"; "As we advance progress against the goals above, we also will continue to purchase 100% RSPO-certified palm oil." *Palm kernel oil: "Establish traceability to palm kernel oil mills by December 31, 2015. (Achieved)"; "Invest in and work with palm kernel oil suppliers, and the smallholders who supply them, with the aim of improving both practices and livelihoods to establish zero deforestation in our supply chain by 2020." *Palm oil derivatives and palm kernel oil derivatives: "Require suppliers to put forward time-bound plans by end of 2016 to ensure no deforestation in their supply chains." | 2016 Sustainability Report |
| 88 | BASF | Preserving ecosystems: "Our production sites reviewed for proximity to internationally protected areas; "BASF partnership supports preservation of biodiversity" *Renewable resources: "Bio-based PolyTHF® 1000 offered for testing purposes for the first time"; "New voluntary commitment and goals for procuring palm oil products" | 2015 Economic, environmental and social performance |
| 89 | Engie | Managing natural resources in a sustainable manner: "To ensure the sustainability of its activities, ENGIE is implementing measures in order to improve its management of water resources, and to reduce the impact of its activities on biodiversity." | 2016 Integrated Report |
| 97 | Target | *Planet: Driving Positive Impact: "Support responsibly managed forests and palm oil production"; "Champion responsibly grown and harvested cotton and ensure that it’s used in our owned-brand products" | 2016 Target Corporate Social Responsibility Report |
| 98 | Pemex | "ENVIRONMENTAL CONSERVATION AND RESTORATION: "Through joint efforts by the company and civil society organizations, we were able to build strategic alliances that have resulted in the development of forest restoration and conservation projects in oil regions.... By supporting these forest restoration and conservation projects, the company: Compensates for the environmental impacts of its operation; Contributes to the conservation of ecosystem services in oil regions; Promotes environmental education; Supports production projects; Contributes to the conservation of biodiversity in oil regions." | 2013 Sustainability Report (latest available in English) |

**Appendix S4.** Examples of biodiversity related activities disclosed in sustainability reports by the 2016 Fortune 100 Global companies (see SI Table 1 for links to each sustainability report). Disclosure of activities was either qualitative (descriptive text in sustainability reports), or quantitative (performance metrics presented in supporting tables or figures in sustainability reports).

| **Activity type** | **Examples of qualitative disclosure** | **Examples of quantitative disclosure** |
| --- | --- | --- |
| ***Management of impacts on biodiversity*** | | |
| Management approach implemented (e.g. reporting against GRI G4-DMA, to demonstrate that a management approach / policy / strategy / action plan has been implemented to manage environmental impacts) | Société Générale: “Regarding biodiversity, Société Générale adopted a specific E&S policy in 2012, which applies to all Group banking and financial operations through review procedures conducted on specific transactions and customers. The E&S General Guidelines also list the UNESCO World Heritage Convention among the international conventions with which the bank undertakes to comply. Lastly, the normative framework of the Equator Principles includes specific standards on biodiversity.” (2016 Corporate Social Responsibility Report, p8)  BP: "Project screening and impact assessment: We evaluate all new projects to determine whether planned activities could affect international protected areas." (2016 Sustainability Report, p39) | Enegie: % of sensitive sites in the European Union with a biodiversity action plan (2016 Integrated Report, page v).  No other examples provided, as only 1 company made a quantitative disclosure here. |
| Environmental impacts managed (e.g., reporting against GRI G4-EN12: Description of significant impacts of activities, products, and services on biodiversity in protected areas and areas of high biodiversity value outside protected areas). | ENI: "Integrated impact assessment: Environmental, Social & Health Impact Assessment (ESHIA) in all new projects" (2016 Sustainability Report, p32)  Citigroup: “MIMIZING IMPACTS TO HABITATS AND COMMUNITIES IN GUATEMALA: Regarding environmental impacts, while the project was designed in compliance with national laws, it required additional biodiversity assessment and mitigation work to comply with IFC [International Finance Corporation] Performance Standards.... Citi and EDC’s ESRM teams required the company to hire an independent biodiversity expert to work with them to bring the project into compliance with IFC standards. This work included developing and implementing a formal biodiversity management plan to avoid impacts to critical habitat and mitigate impacts to sensitive species..." (2016 Global Citizenship Report, p43) | Glencore: Areas owned, leased, managed, located in, adjacent to, or that contain protected areas and areas of high biodiversity value outside protected areas (reported in hectares; used to report against G4-EN11); The amount of land disturbed or rehabilitated (Land rehabilitated (ha) vs Land disturbed (ha)) (2016 Sustainability Report Databook, p18-19).  Chevron: Oil spills to land and water (thousands of barrels per year; 2016 Corporate Responsibility Report p15). |
| Natural capital assessment of impacts and dependencies (e.g., description of how a company has conducted a natural capital assessment of impacts & dependencies) | Hitachi: “Corporate Relationship with Ecosystems: Corporations depend on “ecosystem services,” including the natural supply of materials like fibers and wood, and the ability of ecosystems to maintain the quality and quantity of air, water, and soil. Contributing to ecosystem preservation through both business and social activities enables companies to continue receiving these benefits and to restore ecosystems. Through its business activities, Hitachi is promoting designs and production methods that reduce the impact on ecosystems throughout the product life cycle.” (2016 Sustainability Report, p92).  Nestlé: “We engage with several initiatives that aim to help businesses assess, measure and value their dependencies and impacts. These initiatives aim to enable organisations to better understand their impact and identify appropriate environmental protection, management and restoration projects. Nestlé is an active member of the Cambridge Natural Capital Leaders Program and the WBSC work on redefining value. Nestlé and Nespresso also helped the Natural Capital Coalition pilot its Natural Capital Protocol, a global multi-stakeholder programme designed to support organisations in their understanding and valuation of their own impacts and dependencies on nature. The Natural Capital Protocol was launched in 2016.” (2016 Corporate Social Responsibility Report, p131). | No examples provided, as no companies made a quantitative disclosure relating to natural capital assessments. |
| ***Prevention of impacts*** | |  |
| Sustainable sourcing of natural resources (e.g., timber, palm oil, seafood; including ensuring raw materials are certified as sustainably sourced) | BNP Paribas: "by opting for responsibly sourced paper (made from pulp derived from recycled paper or from sustainably managed forests – PEFC or FSC eco-labels), BNP Paribas helps to protect forest ecosystems and their biodiversity." (2016 Registration Document, p516).  BASF: “In 2015, we completed our joint project with Cargill and the German governmental agency for international cooperation on the sustainable production of coconut oil in the Philippines. Small farmers now produce the world’s first Rainforest Alliance-certified dried coconut meat (copra), from which the oil is extracted." (2015 Economic, environmental and social performance, p96). | Carrefour: Millions of Euros in sales of MSC, ASC and CQL certified seafood (reported in millions of Euros, and the percentage increase from 2015 sales; 2016 Annual Financial Report, p5).  Hitachi: “Design - Products that take ecosystems into consideration” and “Procurement - Encouraging and educating suppliers regarding initiatives to preserve ecosystems” (reported as a percentage of the Business Sites that Enhanced Initiatives Over 3-Year Period; 2016 Sustainability Report, p92). |
| Avoidance of Protected Areas (e.g., reporting against GRI G4-EN11: Operational sites owned, leased, managed in, or adjacent to, protected areas and areas of high biodiversity value outside protected areas) | Exxon Mobil: “"In 2016, an estimated 25 percent of our major operating facilities were within 5 kilometers of designated environmentally sensitive areas” (2016 Exxon Mobil Corporate Citizenship Report, p26).  ENI: "Use of the UNEP-WCMC’s IBAT tool to map the position of protected areas and the presence of species at risk of extinction. IBAT is used to identify the priority operating sites where action plans need to be developed" (2016 Sustainability Report, p39). | Glencore: Areas owned, leased, managed, located in, adjacent to, or that contain protected areas and areas of high biodiversity value outside protected areas (reported in hectares; used to report against G4-EN11; 2016 Sustainability Report Databook, p18-19).  Volkswagen: Plants in the vicinity of nature conservation areas (reported as distance to conservation area (km) and plant area (ha); 2016 Sustainability Report, p120). |
| Avoidance of threatened species (e.g., reporting against GRI G4-EN14: Total number of IUCN Red List species and national conservation list species with habitats in areas affected by operations, by level of extinction risk) | Target: "To the best of our knowledge, Target does not adversely affect the well-being of endangered species because stores and supporting facilities are not constructed in critical habitat areas. Constructed wetlands and ponds have the potential to become habitats for endangered species, or stopover points for migratory birds. Target does not have data for these types of environments at this time." (2016 Target Corporate Social Responsibility Report, p43).  Exxon Mobile: "Our approach to managing biodiversity and ecosystem services recognizes several factors, including the rarity of individual species, their roles in different ecosystems and habitats, their vulnerabilities and their cultural significance." (2016 Corporate Citizenship Report, p26). | Enel: The number of IUCN Red List species affected by projects in different countries (reported as the number of species in threatened and low risk categories; 2016 Sustainability Report, p182 – 183).  Petrobras: The number of threatened species (International list (IUCN) and National listed species) inside the influence of Petrobras activities (reported as the number of vulnerable, endangered and critically endangered species; 2016 Sustainability Report, p57). |

| **Disclosure type** | **Examples of qualitative disclosure** | **Examples of quantitative disclosure** |
| --- | --- | --- |
| ***Protection or restoration*** | |  |
| Habitats protected or restored. (e.g., GRI G4-EN13: Habitats protected or restored) | Dailmer: “"An environmental protection zone in a semi-arid landscape. Daimler has cooperated with biologists from the Autonomous University of Nuevo León to create a botanical garden at the edge of the grounds of the Mercedes-Benz bus plant in Monterrey, Mexico. For the facility’s employees, the garden is an oasis of peace and relaxation. Above all, this ecological reserve, which covers about two square kilometers, is helping to preserve indigenous animals and vegetation.” (2016 Sustainability Report, p72).  E.ON: “I boschi E.ON” (E.ON Woods) now consist of more than 28,000 trees in nine areas around Italy. E.ON Italia aims to reach 60,000 trees by the end of 2017, making E.ON Woods one of Italy’s biggest reforestation projects.” (2016 Sustainability Report, p111). | Hitachi: New ecosystem preservation activities implemented (reported as the number of activities implemented) (2016 Sustainability Report, p71).  Volkswagen: Environmental protection costs for the German locations of Volkswagen (reported as € million/year) (2016 Sustainability Report, p119). |
| ***Monitoring*** | |  |
| Monitoring of impacts or restorations. Monitoring is being undertaken to evaluate impacts or restorations | Honda: "we cooperate with “Monitoring Sites 1000” (a project for promoting the monitoring of survey sites of important ecosystems) implemented by the Japanese government as a member of the International Union for Conservation of Nature and Natural Resources (IUCN), which creates an annual Red List. For the above program we continue to carry out fixed-point observation and reporting on ecosystems." (2016 Honda Sustainability Report, p 36).  Gazprom: “Fauna studies near the Prirazlomnaya Platform: Gazprom Neft Shelf conducted a study during the reporting year on how a wide range of fauna species could be potentially impacted by the Prirazlomnaya platform in the Pechora Sea as well as escort vessels and tankers that deliver oil to Murmansk from the Novoportovskoye and Prirazlomnoye fields based on a programme and list of indicator species. The research was conducted offshore, on the coast and in coastal waters." (2016 Sustainable Development Report, p105). | Petrobras: The distribution of studies and projects carried out on biodiversity (reported as a percentage of different types of projects, e.g., environmental risk evaluation, wildlife management and environmental monitoring) (2016 Sustainability Report, p58).  No other examples provided, as only 1 company made a quantitative disclosure in relation to monitoring. |

| **Disclosure type** | **Examples of qualitative disclosure** | **Examples of quantitative disclosure** |
| --- | --- | --- |
| ***Engagement*** | |  |
| Staff & supply chain engagement (e.g., communication about biodiversity, or implementation of conservation volunteer programs) | Samsung Electronics: "We continuously monitor water quality and the aquatic ecosystems of streams where our wastewater is released, and regularly conduct conservation activities for stream ecosystems together with local NGOs, family members of employees, and students of local schools… Samsung shall consistently communicate with our employees, local communities, NGOs, and stakeholders, and contribute to promoting the biodiversity conservation activities of local communities." (2016 Sustainability Report, p182).  General Motors: "Support Biodiversity at GM Sites:… In 2016, we earned the following national awards: the WHC’s Corporate Conservation Leadership Award, Employee Engagement Award and the Formal Learning Project Award for the habitat at our Langley (Vancouver) Parts Distribution Centre…” (2016 Sustainability Report, p150). | No examples provided, as no companies made a quantitative disclosure relating to staff and supply chain engagement. |
| External partnerships established. Description of partnerships established with NGOs, government agencies, or industry groups | Walmart: “Walmart, through collaboration with the “National Fish and Wildlife Foundation (NFWF), in 2005 Walmart helped establish the Acres for America program” (2016 Walmart Global Responsibility Report, p86).  Shell 2016 Sustainability Report: "Shell has environmental partnerships with the International Union for Conservation of Nature (IUCN), Wetlands International, The Nature Conservancy and Earthwatch. Our environmental partners can bring specific expertise to our projects in areas such as biodiversity, while at the same time advancing their own scientific or conservation knowledge by working on our projects." (2016 Global Responsibility Report, p58). | No examples provided, as no companies made a quantitative disclosure relating to external partnerships. |
| Community engagement (e.g., community led environmental programs) | Walmart: “Restored urban lands and habitats and connected youth to the outdoors by investing more than $1.5 million in community-based projects located in New York, Chicago, Los Angeles, San Diego, Denver, Bridgeport, Portland (Ore.), Albuquerque and Washington, D.C.” (2016 Walmart Global Responsibility Report, p86).  Électricité de France: “Training and raising awareness of the company’s employees are important levers for progress with regard to taking into account biodiversity issues challenges across the whole value chain. In France, eight business guides have been published, written in a manner which very closely reflects the biodiversity issues and challenges species to each operational activity. In 2016, EDF Énergies Nouvelles issued a “Biodiversity and Energy Wind power and Solar power” guide for its employees. A “Biodiversity and Electricity Grids” guide has also been made available to the entities and subsidiaries the most concerned. Training and raising awareness of the company’s employees are important levers for progress with regard to taking into account biodiversity issues challenges across the whole value chain. Several internal training courses on biodiversity are offered to employees. In 2016, 160 employees took these training courses.” (2016 Reference Document, p174). | Toyota: Toyota Environmental Activities Grant Program – grants provided “to support the environmental activities of NPOs and other groups with the aim of solving issues in environmental fields and supporting the development of the next generation of human resources” (reported as the number of projects supported in regions around the world per financial year) (Sustainability Data Book 2016, p91).  No other examples provided, as only 1 company made a quantitative disclosure in relation to community engagement. |

| **Disclosure type** | **Examples of qualitative disclosure** | **Examples of quantitative disclosure** |
| --- | --- | --- |
| ***Investment in conservation action or research*** | |  |
| Supporting research. Description of external research that is supported and/or invested in (e.g., by universities and NGOs) | Gazprom: “The Company develops environmental programmes and introduces new technologies in cooperation with scientific institutions and non-governmental organisations. In particular, Gazprom Neft is drafting and implementing biodiversity conservation programmes jointly with the Severtsov Institute of Ecology and Evolution. Gazprom Neft cooperated with the Sysin Research Institute of Human Ecology and Environmental Health over the last year to develop unified pollutant standards to be used by industrial enterprises throughout Russia.” (2016 Sustainable Development Report, p87).  Royal Dutch Shell: "We are also working with scientists to explore the depths of the ocean. " (2016 Sustainability Report, p44). | No examples provided, as no companies made a quantitative disclosure relating to research supported. |
| Investment in conservation action  Description of conservation programmes that have been invested in (e.g., financing conservation projects - separate to partnerships) | Enel: “Investments of 369 million euro rose compared to the previous year (+18%) and mainly concerned existing plants. In the environmental sector investments mainly concerned air and climate protection, followed by the protection of biodiversity and countryside.” (2016 Sustainability Report, p172)  Total: "For the period of 2013-2017, the Group has renewed its commitment to its foundation, which has a five-year budget of €50 million. The Total Foundation is active in four fields: health, solidarity, oceans and marine biodiversity, culture and heritage." (p166, 2016 Registration Document) | No examples provided, as no companies made a quantitative disclosure relating to investment in conservation action. |

**Supporting Information: Literature cited**

F&C. 2004. Is biodiversity a material risk for companies? An assessment of the exposure of FTSE sectors to biodiversity risk. F&C Asset Management. UK.

Fortune. 2016. The Fortune 500 Global Companies. Website: <http://beta.fortune.com/global500/>. Accesed: 01 August 2016
